# Intramolecular isotopic evidence for bacterial oxidation of propane in subsurface natural gas reservoirs

**DOI:** 10.1073/pnas.1817784116

**Published:** 2019-03-18

**Authors:** Alexis Gilbert, Barbara Sherwood Lollar, Florin Musat, Thomas Giunta, Songcan Chen, Yuki Kajimoto, Keita Yamada, Christopher J. Boreham, Naohiro Yoshida, Yuichiro Ueno

**Affiliations:** ^a^Department of Earth and Planetary Sciences, Tokyo Institute of Technology, Meguro, 152-8551 Tokyo, Japan;; ^b^Earth-Life Science Institute, Tokyo Institute of Technology, Meguro, 152-8550 Tokyo, Japan;; ^c^Department of Earth Sciences, University of Toronto, Toronto, ON, Canada M5S 3B1;; ^d^Helmholtz Centre for Environmental Research – UFZ, 04318 Leipzig, Germany;; ^e^Department of Environmental Chemistry and Engineering, Tokyo Institute of Technology, Yokohama, 226-8503 Kanagawa, Japan;; ^f^Geoscience Australia, Canberra, ACT 2601, Australia

**Keywords:** intramolecular isotope, biodegradation, hydrocarbons, propane

## Abstract

Microbial anaerobic oxidation of hydrocarbons is a key process potentially involved in a myriad of geological and biochemical environments yet has remained notoriously difficult to identify and quantify in natural environments. We performed position-specific carbon isotope analysis of propane from cracking and incubation experiments. Anaerobic bacterial oxidation of propane leads to a pronounced and previously unidentified ^13^C enrichment in the central position of propane, which contrasts with the isotope signature associated with the thermogenic process. This distinctive signature allows the detection and quantification of anaerobic oxidation of hydrocarbons in diverse natural gas reservoirs and suggests that this process may be more widespread than previously thought. Position-specific isotope analysis can elucidate the fate of natural gas hydrocarbons and provide insight into a major but previously cryptic process controlling the biogeochemical cycling of globally significant greenhouse gases.

## Significance

Microorganisms can oxidize hydrocarbons anaerobically, but the detection and quantification of this process in natural settings remains difficult, impeding reliable estimation of these processes at the global scale. We have used the technique of position-specific isotope analysis of propane and show that anaerobic biological degradation of propane leads to a specific signature that differs from that of propane produced from thermal decomposition of higher hydrocarbons. When applied to natural gas reservoirs, we show that anaerobic bacterial oxidation of propane can be detected and quantified, which is not the case with the use of conventional methods. Our findings are thus of importance for the detection of subsurface biology, for the understanding of the carbon cycle, and more broadly for environmental sciences.

Understanding the sources and sinks of nonmethane hydrocarbons (NMHCs) is of key importance for investigations of atmospheric chemistry, energy resources, and the global C-cycle. While their global warming potential as greenhouse gases is less than that of methane, NMHCs influence the global radiative balance indirectly by producing ozone in the troposphere and by reacting with OH radicals, their primary atmospheric sink (1, 2). Furthermore, NMHCs are consistently associated with methane, a strong greenhouse gas. The global emissions of ethane and propane, the most abundant NMHCs in the atmosphere, are estimated to be about 15 Tg/y (3), although recent studies suggest that this number may be underestimated by a factor of 2 or 3 (4). Recent studies have highlighted the need for a much better understanding of the sources and sinks of NMHCs, and investigation of their global budget is still an active area of research (2–6).

NMHCs form through the thermal decomposition of organic matter in sedimentary basins and are brought to the atmosphere by direct emissions from fossil fuel usage, biomass burning, or natural seepages (3, 4). During their transition through sediment or water columns, NMHCs are used as potent growth substrates by microorganisms, which thereby act as filters altering the amounts released to the atmosphere (7). Given the anoxic nature of natural gas reservoirs, anaerobic oxidation of hydrocarbons (AOH) is of critical importance to understand the processes controlling hydrocarbon sinks and biogeochemical cycles catalyzed by microorganisms in the subsurface. AOH proceeds using nitrate, sulfate, or iron as electron acceptors or under methanogenic conditions (7), thus potentially affecting not only the carbon but also the sulfur, nitrogen, and metal cycles. Oxidation of hydrocarbons in anoxic environments was considered unlikely until the 1990s when a sulfate-reducing bacterium growing on hexadecane as the sole carbon source was isolated for the first time (7, 8). The first microbial strain able to metabolize natural gas NMHCs under anoxic conditions was isolated from a hydrocarbon seep area in Guayamas Basin (9)—a sulfate-reducing Deltaproteobacterium affiliated with the Desulfobacteraceae family that uses sulfate as electron acceptor. This organism can use propane and *n*-butane as the carbon source, while both shorter (ethane) or longer alkanes (*n*-pentane) are not metabolized (9). While progress in the isolation of microorganisms clearly shows that NMHCs can be oxidized anaerobically, identifying and especially quantifying AOH processes in natural environments remains a significant challenge (10–12). Confirming AOH activity and evaluating its impact on a hydrocarbon reservoir typically requires multiple lines of chemical, isotopic, and microbiological evidence. One key line of evidence for AOH is commonly inferred through natural abundance hydrogen and carbon isotope analysis of hydrocarbon compounds (10, 12). Due to kinetic isotope effects, microorganisms tend to preferentially use lighter isotopes and thus the residual pool of hydrocarbons becomes enriched in the heavier isotopes (^13^C and ^2^H). Consequently, the isotope composition of remaining hydrocarbons becomes less negative as the extent of biodegradation increases. Importantly, however, ^13^C and ^2^H abundances also vary with maturity and source of the gas, as well as with the formation temperature (13), leading to difficulties in distinguishing biodegradation from thermogenic processes. Therefore, while anaerobic oxidation can be a major process controlling hydrocarbon sinks, it is difficult to identify except in rare cases where the bulk of the hydrocarbon pool has been largely consumed (10–12). Global mass balance budgets and flux estimates for hydrocarbon cycling in fields as wide-ranging as petroleum exploration, greenhouse gas emissions, fugitive gas leakages, geomicrobiology, and oceanography require a more sensitive and reliable means of identifying and quantifying the process of AOH in situ. The present work takes advantage of recent development in position-specific isotope analysis of propane (14) to detect and quantify the AOH process in natural environments.

## Results

### Intramolecular Isotopic Signature of Thermogenic Propane.

**Fig. 1.**
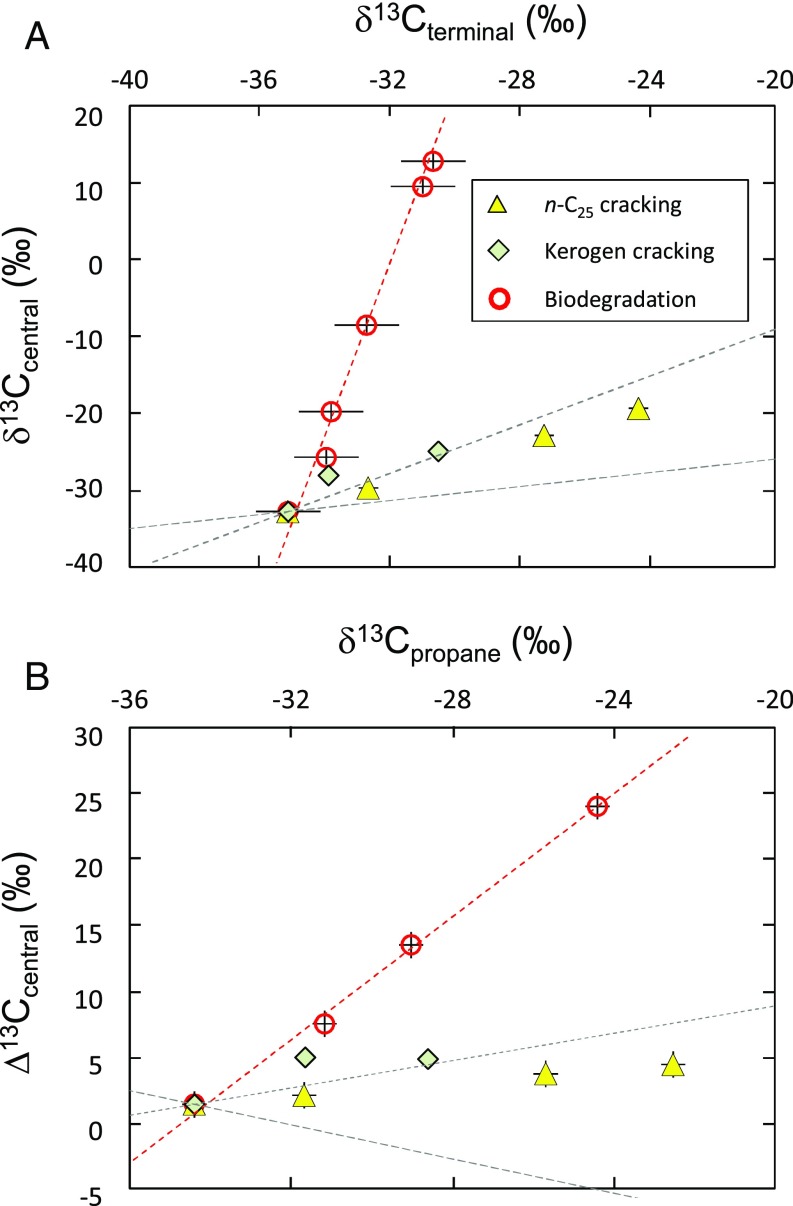
δ^13^C_terminal_ as a function of δ^13^C_central_ (*A*) and bulk isotope composition (δ^13^C_propane_) as a function of Δ^13^C_central_ (*B*) for propane from experiments simulating propane formation via thermogenic cracking and via biodegradation of propane. For sake of clarity, all points are normalized to start from propane with similar carbon isotope composition. Red circles: biodegradation of propane by *Desulfosarcina* strain BuS5 (red dotted line: fitted linear trend for biodegradation experiments). Yellow triangles: thermogenic cracking of long-chain alkane *n*-C25 at 500 °C for different times (0.5, 1, 2, and 5 h). Green diamonds: Thermogenic cracking of kerogen at different temperatures [330, 360, and 390 °C; from Piasecki et al. (17)]. The long gray dashed line is the theoretical slope obtained from calculations of isotope fractionation factors associated with thermal formation of propane through the thermal cracking of a longer chain alkane (primary cracking). The gray dashed line is the theoretical slope obtained from calculations of isotope fractionation factors associated with thermal degradation of propane (secondary cracking).

With a few exceptions (15), NMHCs in natural gas are considered to be thermogenic in origin, that is, arising from the thermocatalytic cracking of sedimentary organic matter (16). Variations in δ^13^C values of NMHCs are thus related to the extent and temperature at which natural gas formed (13). Thermogenic generation is associated with a normal carbon isotope effect (^12^k/^13^k > 1), and hence the propane formed has more negative δ^13^C values compared with the source organic matter. Following a Rayleigh fractionation process, the δ^13^C value of propane increases as maturity increases (13). We conducted experiments to determine position-specific carbon isotope fractionation factors associated with thermogenic propane formation. The experiments involved the cracking of *n*-C_25_ at 500 °C and measuring the bulk and position-specific carbon isotope composition of propane generated at different times. The δ^13^C values of propane increase with the extent of thermal cracking, with values ranging from −34.4‰ to −22.5‰. At the position-specific level, the isotopic ratio of central carbon in a propane molecule (δ^13^C_central_) increases by 13.9‰, while that of terminal carbon (δ^13^C_terminal_) increases by 10.9‰, corresponding to a relative ^13^C enrichment at the central position Δ^13^C_central_ (= δ^13^C_central_ − δ^13^C_terminal_) of 3‰ (Fig. 1*B*). Our data are consistent with those recently obtained from kerogen cracking (17) but differ slightly from model-derived data for which fractionation was predicted to occur mainly on the terminal position of propane (13). According to current models, cracking of a precursor (e.g., a long-chain alkane) leads to a primary isotope effect associated with the C–C bond breaking, which is predicted to lead to ^13^C depletion at one of the terminal position of propane, the other positions being unaffected (13, 18). As a result of Rayleigh fractionation, as the reaction progresses the δ^13^C value of the terminal position of propane is predicted to move toward that of the starting precursor. During that process, the δ^13^C value of the central position is assumed to be affected only by secondary isotope effects, which are generally one order of magnitude smaller than primary isotope effects (13). Our data show that in fact the δ^13^C value of the central position is also affected by the thermogenic process, to an extent that exceeds secondary carbon isotope fractionation. We propose that secondary cracking of propane occurs during the thermogenic process and must be taken into account when considering isotope fractionation associated with natural gas formation. Indeed, toward the end of the gas window, NMHCs are eventually cracked themselves, leading to so-called dry gas where methane is virtually the only hydrocarbon remaining. Theoretical calculations of propane thermal degradation suggest that both positions are affected (13), leading to a trend similar to that obtained from cracking experiments (Fig. 1).

### Intramolecular Isotopic Signature of Biodegraded Propane.

**Fig. 2.**
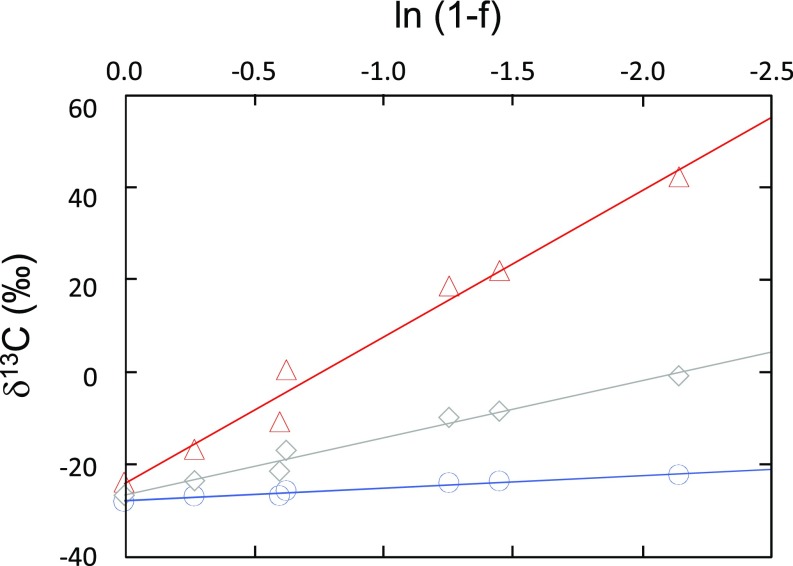
Isotopic composition of bulk propane (gray diamonds), terminal position (blue circles), and central position (red triangles) of the remaining propane (1 − f) during the course of anaerobic propane oxidation by the sulfate-reducing bacterium *Desulfosarcina* sp. strain BuS5. Fractionation factors (ε, ‰) are calculated according to the slope of the line. ε_bulk_ = 12.8‰ ± 0.8‰; ε_terminal_ = 2.8‰ ± 0.2‰; ε_central_ = 33‰ ± 2‰. Error bars are smaller than the plotted symbols.

To elucidate biological processes of propane cycling as well, this study also measured position-specific isotope fractionation associated with bacterial anaerobic oxidation of propane. Sulfate-reducing bacteria activate propane and *n*-butane by generating an alkyl radical which reacts with the double bond of fumarate acting as cosubstrate, eventually leading to the formation of alkylsuccinates (7, 9). The activation reaction is catalyzed by alkylsuccinate synthases, glycyl radical enzymes which are common activating enzymes for anaerobic hydrocarbon oxidation (7). Previous studies have shown that propane is activated selectively at the central position (70% for central vs. 30% for terminal) (19), as formation of a radical at the CH_2_ position is energetically favored compared with the CH_3_ position (20). We measured ^13^C position-specific isotope fractionation factors associated with propane oxidation by *Desulfosarcina* sp. strain BuS5. With respect to traditional whole-molecule δ^13^C values, the remaining bulk propane is enriched by a factor ε_propane_ = 12.8 ± 0.8‰, a range similar to that measured previously (19). Importantly, in this study we found that for the central position the fractionation factor is one order of magnitude higher (ε_central_ = 33 ± 2‰) than for the terminal position (ε_terminal_ = 2.8‰ ± 0.2) (Fig. 2). Calculations from isotopic fractionation factors obtained here indicate that the activation occurs at 85% on the central position of propane, which is consistent with previous findings using ^2^H-labeled propane (19). As a consequence, during bacterial anaerobic oxidation of propane, the residual propane will be ^13^C-enriched mainly at the central position, while the terminal position will be barely affected.

Most importantly, this study demonstrates that the fractionation associated with the central and the terminal positions describe distinctly different slopes for thermogenic propane, and for microbially oxidized propane (Fig. 1), providing a clear signal for detection and quantification of AOH in natural environments.

### Application to Natural Gas Reservoirs.

**Fig. 3.**
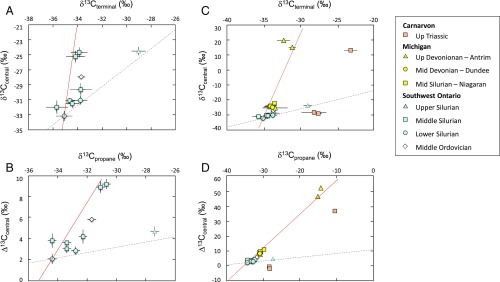
Bulk (δ^13^C_propane_) and position-specific carbon isotope composition of terminal (δ^13^C_terminal_) and central (δ^13^C_central_) positions of propane from Southern Ontario, Michigan, and Northern Carnarvon Basins. *A* and *B* show only data from Southwest Ontario, while *C* and *D* show the expanded scale and additional data from other basins. Δ^13^C_central_ is the relative ^13^C enrichment at the central position (Δ^13^C_central_ = δ^13^C_central_ − δ^13^C_terminal_). Lines are based on the slopes calculated from experiments and start from the propane sample with the lowest δ^13^C and Δ^13^C_central_ values. Red line: biodegradation trend identified in Fig. 1 *A* and *B*; gray dotted line: thermogenic trend from cracking experiments of an *n*-alkane (yellow triangles in Fig. 1 *A* and *B*). *A* and *B* are extended parts of *C* and *D*.

To characterize AOH in real geological settings, we analyzed samples from different natural gas reservoirs: Southwest Ontario Basin (Canada), Michigan Basin (United States), and Northern Carnarvon Basin (Australia) (*SI Appendix*, Fig. S1). Based on chemical and isotope composition, hydrocarbons in Southwest Ontario are suggested to be mainly thermogenic with locally significant components of microbial methane (up to 40−50%; *SI Appendix*, Fig. S2), consistent with previous observations (21, 22). Results of propane from Southwest Ontario in this study show that, except for one sample, there is a pronounced ^13^C enrichment of the central position of propane: δ^13^C_central_ increases by 9‰ while δ^13^C_terminal_ increases by 2‰ (Fig. 3*A*). This leads to an increase in the relative ^13^C content in the central position of propane Δ^13^C_central_ as δ^13^C_propane_ values increase (Fig. 3*B*). The selective ^13^C enrichment of the central position fits well with the slope defined for propane-degrading bacteria in this study (Fig. 1), suggesting the occurrence of bacterial oxidation of propane in Southwest Ontario strata. As stated above, one of the samples analyzed from the upper Silurian does not fit the biodegradation trend (Fig. 3), but we note it does fit the thermogenic trend obtained from cracking experiments. Whether this sample arises from the same source with a different maturity or from another source remains to be elucidated. On the whole, our results suggest a biodegradation sequence where propane is oxidized to different extents across the Southwest Ontario stratigraphic formations. The sample with the lowest δ^13^C values can be considered as the “starting” propane, namely the least biodegraded. Any biodegraded propane sample must then fit on the biodegradation line, at a distance to the starting point depending on the extent of biodegradation. The data from Southern Ontario represents a typical case where molecular variation of isotope composition of propane alone (within 3‰; Fig. 3*B*) would not allow the detection of AOH. A first approximation based on the fractionation factors calculated here suggests for the most extreme samples (highest Δ^13^C values) that degradation of up to 20% of the propane has occurred—demonstrating the ability of this approach to identify the effects of AOH much more effectively that traditional approaches which provide a strong signal of this important process only once the majority (typically >80 to 90%) of the hydrocarbon pool has been degraded.

Despite representing important hydrocarbon resources (23), it can be noted that the source and the origin of thermogenic gases in Southwest Ontario has always been ambiguous. The fact that these Paleozoic strata, situated on the Algonquin Arch, have presumably always remained immature to moderately mature (24) has led several authors to consider an external origin for hydrocarbons, for instance by suggesting that they could have migrated from the adjacent Michigan or Appalachian Basins (21, 22). The exact timing and onset of microbial activity that added a microbial component to these gases is still an active subject of research which is beyond the scope of this study. The role of microbial hydrocarbon cycling in the Southwest Ontario strata has been supported by previous studies based on many other lines of evidence. Specifically, the presence of sulfate-reducing bacteria in the Southwest Ontario formations has been suggested based on genomic analysis and the presence of pyrite in some Ordovician strata (22). Biological activity is further supported by the presence of microbially produced methane in these formations, implying the presence of methanogens (21, 22). While the salinity of the lower Silurian and Ordovician units is high in the present day (total salinity exceeding 250 g/L) (22, 25) and may exceed the typical limits for bacterial growth, methanogenesis is suggested to have been active at a time when the salinity was lower (22), which could also apply to AOH. Further, some sulfate-reducing bacteria are known to be tolerant to conditions similar to those observed in Southwest Ontario Basin (26).

Results from the Michigan Basin show an even more pronounced trend (Fig. 3) with a ^13^C enrichment on the central position of propane of *ca*. 47‰ between different samples from upper Devonian (Antrim Shale), leading to δ^13^C values for the central position of up to +21‰. AOH in Michigan Basin was first suggested by Martini et al. (27) based on the carbon isotope composition of ethane and propane. In contrast to Southwest Ontario basin, the microbial activity in the Antrim shale is suggested to have been triggered by freshwater recharges during the Pleistocene (27, 28). Sulfate-reducing bacteria were later identified in the Antrim shale, building a strong case for the role of hydrocarbon anaerobic oxidizing bacteria, although a direct link to NMHC oxidation could not be definitely established (27). Here we show a clear AOH trend which corresponds to an oxidation of propane of *ca*. 75%. Interestingly, recent results from clumped isotopes of methane suggest the involvement of anaerobic oxidation of methane in Michigan Basin as well (28). While methane and propane oxidation are not conducted by the same microorganisms, they both use the same electron acceptor, sulfate, and may likely be found in similar environments. The relative rate of methane and propane oxidation will then be driven by the competition for sulfate.

The Northern Carnarvon Basin is a third basin in which propane and *n*-butane have been previously suggested to experience microbial oxidation based on their ^13^C and ^2^H enrichments (29). Here we show a similar trend for Northern Carnarvon Basin samples comparable to that for Michigan Basin samples (Fig. 3), indicating an extent of propane oxidation of *ca*. 75%. Interestingly, while the trend is similar to the Antrim shale, the δ^13^C values of the original (i.e., nonoxidized) propane are different for both basins. In particular, the terminal position is 7 to 8‰ richer than Michigan and Southwest Ontario propane. Variations of δ^13^C of natural gas hydrocarbons are governed by parameters including the carbon source, the degree of maturity, and the accumulation history of the gas. All these parameters may influence propane’s carbon isotope composition at the position-specific level, leading to propane samples with distinct carbon isotope patterns for different basins, as already observed from recent measurements (17, 30). This further emphasizes the significance of the position-specific isotopic trends for thermogenic versus AOH affected propane identified in this study, as the slopes of the trend lines are independent of the original δ^13^C value of the propane source and hence provide insight into processes of AOH independent of the variations in source signature.

The occurrence of AOH in the natural gas reservoirs studied here is further supported by the trend of propane concentration: For all field samples in this study, a large ^13^C enrichment on the central position (i.e., presumably highly biodegraded) correlates with lower propane concentration (*SI Appendix*, Fig. S3). Interestingly, the correlation of Δ^13^C_central_ with concentration is also observed for *n*-butane, but not for methane and ethane, which are not metabolized (31), further supporting the involvement of AOH in the reservoirs. The correlation between propane and *n*-butane concentrations and Δ^13^C_central_ is dependent on the fractionation factor, itself dependent upon environmental and physiological factors (32). Further experiments with additional strains and under a range of environmentally relevant conditions would be the goal of future studies and enhance the ability to use these signatures to quantify the effects of AOH.

## Conclusion

The key result in this paper is the demonstration of the carbon isotopic enrichment trend for the central and terminal positions and the distinct differences in slope that can be used to identify the effects of AOH in an in situ real-world setting. Overall, anaerobic oxidation of propane leads to a trend that is reproducible between incubation experiments and natural environments, allowing the identification and quantification of AOH in natural environments. This study provides not only insight into hitherto cryptic pathways in the hydrocarbon cycle but also an essential input for the development of global mass balance and flux models estimating the global significance of the process in natural environments.

## Methods

### Sample Location and Sampling.

The Southwest Ontario sedimentary succession is composed of late Cambrian- to Devonian-age sediments (21). The samples analyzed in this study were from Silurian and middle Ordovician and were collected from June 2012 to December 2013. Gases were sampled from commercial wellheads and collected in preevacuated borosilicate 160-mL glass vials fixed with 50 μL saturated HgCl_2_ and sealed with blue butyl rubber stoppers as described in Ward et al. (33). Gas from the wellhead was introduced into a large reservoir in which the gas was allowed to flow for about 10 min. Gas in the reservoir was then sampled with a gas-tight syringe and injected into the vial. For a complete description of the basin and sampling method see ref. 28.

The Michigan Basin is located in Michigan and is composed of sediments ranging from Cambrian- to Pennsylvanian-age. Samples analyzed here are from Devonian and Silurian strata. They were collected in refrigeration-grade copper tubes (60 cm long, 10 mm in diameter), using the procedure developed in ref. 34. For a complete description of the basin and sampling method see ref. 28.

The Northern Carnarvon Basin is located off the coast of western Australia and contains up to 15 km of largely Mesozoic sedimentary rocks (35). Samples from the Northern Carnarvon Basin were from Middle Triassic to Early Cretaceous reservoirs and were collected by the operator between 2000 and 2003 into preevacuated stainless cylinders (150-mL capacity) at reservoir pressure. Gases were subsampled into cold-sealed copper tubes (15-inch length × 3/8-inch o.d.) at 30 psi for transport before analysis.

### Determination of the δ^13^C Value of Hydrocarbons.

The ^13^C composition of hydrocarbons (methane, ethane, propane, *i*-butane, and *n*-butane) was determined using a gas chromatograph coupled with an isotope-ratio mass spectrometer (IRMS) (Delta^plus^XP; Thermo Fisher Scientific) via a combustion furnace and a conflow interface (GC Combustion III; Thermo Fisher Scientific). High-purity helium was used as the carrier gas. The conditions of the GC oven were as follows: injector temperature 250 °C, split mode (variable split ratio), flow rate 1.5 mL/min, and oven temperature program 50 °C (maintained 1 min) raised to 150 °C (maintained 10 min) at a rate of 10 °C/min. The column used was a HP-PLOT-Q (30-m × 0.32-mm i.d., 10-µm film thickness; Varian). The effluent was then introduced into a combustion furnace (ceramic tube packed with CuO, NiO, and Pt wires, operating at 960 °C) before being analyzed by the IRMS. Isotopic standardization was made by CO_2_ injections calibrated against the National Institute of Standards and Technology (NIST) natural gas standard NGS-2 (36). SDs from three to five measurements were generally lower than 0.3‰.

### Determination of Intramolecular δ^13^C Composition in Propane.

Propane samples were introduced using a gas-tight syringe into an online pyrolysis system coupled with GC-C-IRMS, as previously described in ref. 14. High-purity helium was used as the carrier gas. A first GC column (HP-PLOT-Q, 30-m × 0.32-mm i.d., 10-µm film thickness; Varian) was connected to a high-temperature conversion furnace (deactivated fused-silica capillary column 0.25-mm i.d. inserted in a ceramic tube of 25-cm × 0.5-mm i.d., operating at different temperatures) to pyrolyze propane at a temperature of 850 °C. The pyrolytic fragments were separated on a second GC capillary column (HP-PLOT-Q, 30-m × 0.32-mm i.d., 10-µm film thickness; Varian) and introduced into a combustion furnace (ceramic tube packed with CuO, NiO, and Pt wires, operating at 960 °C) before being analyzed by the IRMS (Delta XP; Thermo Fisher Scientific Inc.). The conditions of the first GC oven were as follows: injector temperature 250 °C, split mode (variable split ratio), flow rate 2.5 mL/min, and oven temperature program 50 °C (15 min) raised to 100 °C (10 min) at a rate of 10 °C/min then raised to 150 °C (15 min) at a rate of 20 °C/min and held at 150 °C for 15 min. The second GC oven was kept at 40 °C throughout the analysis. Once the C_1_ and C_2_ fragments from pyrolysis of hydrocarbons were eluted from the second column the temperature of the second GC oven was raised to 150 °C at 20 °C/min to elute unreacted hydrocarbons. Isotopic standardization was made by CO_2_ injections calibrated against the NIST natural gas standard NGS-2 (34). The connections between the GC columns and the pyrolysis and combustion furnaces were made using a deactivated fused-silica capillary column (0.25-mm i.d.). The relative enrichment in a given position (Δ^13^C_central_, in per mille) is defined as the difference in isotopic composition between central and terminal positions. Three fragments are used for its calculation: CH_4_, C_2_H_4_, and C_2_H_6_. CH_4_ and C_2_H_6_ arise from the terminal position only, while C_2_H_4_ arises from an equal contribution of terminal and central positions. The relative ^13^C enrichment in the central position Δ^13^C_central_ can thus be calculated as follows:

$$\Delta^{13}{\text{C}}_{\mathrm{central}}=-\left(\delta^{13}C_{\mathrm{CH}4,\mathrm{original}}-\delta^{13}C_{C2H4}\right)*2$$ [1]

with

$$\delta^{13}C_{\mathrm{CH}4,\mathrm{original}}=\left(\delta^{13}C_{\mathrm{CH}4}*A_{\mathrm{CH}4}+\delta^{13}C_{C2H6}*A_{\text{C}2H6}\right)/\left(A_{\mathrm{CH}4}+A_{C2H6}\right),$$ [2]

where A is the area of the fragment peaks.

Pyrolysis induces carbon isotope fractionation, leading to fragments being ^13^C-depleted compared with the original propane. This is apparent when δ^13^C values of propane are calculated using values of the fragments δ^13^C′_propane_:

$$\delta^{13}C_{\mathrm{propane}}^{\prime }=\left(\delta^{13}C_{\mathrm{central}}^{\prime }+2*\delta^{13}C_{\mathrm{terminal}}^{\prime }\right)/3$$ [3]

$$\delta^{13}C_{\mathrm{propane}}^{\prime }-\delta^{13}C_{\mathrm{propane}}=\varepsilon .$$ [4]

The isotope fractionation factors ε in the conditions used here is 3.8 ± 0.8‰ and is constant for all samples (14). We then correct position-specific δ^13^C values so that they fulfill the following equation:

$$\delta^{13}C_{\mathrm{propane}}=\left(\delta^{13}C_{\mathrm{central}}+2*\delta^{13}C_{\mathrm{terminal}}\right)/3.$$ [5]

This ends up correcting δ^13^C values by a shift corresponding to ε:

$$\delta^{13}C_{\mathrm{central}}^{\prime }=\delta^{13}C_{\mathrm{central}}+\varepsilon$$ [6]

$$\delta^{13}C_{\mathrm{terminal}}^{\prime }=\delta^{13}C_{\mathrm{terminal}}+\varepsilon .$$ [7]

SDs from three to five measurements were typically lower than 1.0‰.

### Cultivation of *Desulfosarcina* sp. Strain BuS5.

Strain BuS5 was routinely cultivated in 120-mL serum bottles provided with 60 mL of anoxic, bicarbonate-buffered artificial seawater medium as previously described (8). Culture bottles were sealed with butyl-rubber stoppers, under a headspace of N_2_:CO_2_ (9:1). Propane (purity 3.5; Air Liquide) was added to the headspace at a partial pressure of 1 bar (final pressure 2 bar). The bottles were inoculated with 10% vol/vol of a grown culture. For the fractionation experiment, a number of eight serum bottles (60 mL volume) were provided with 30 mL artificial sea water medium, flushed with N_2_:CO_2_ (9:1), closed with butyl-rubber stoppers, and inoculated with 3 mL of a grown culture; 4 mL propane (15% vol/vol headspace) was injected to the headspace, yielding a 15% vol/vol concentration. The bottles were incubated at 28 °C with continuous horizontal shaking (100 rpm). When the cultures reached preestablished sulfide concentrations, corresponding to degradation of ∼0, 20, 40, 50, 60, 70, 80, and 90% of the added propane, they were inactivated by addition of 1 M NaOH to a pH of about 12. Control incubations were prepared by adding 33 mL sterile, artificial sea water medium to serum bottles (60 mL volume); the bottles were flushed with N_2_:CO_2_ (9:1), closed with butyl-rubber stoppers, and provided with 4 mL propane. Controls were inactivated in a similar way as the cultures. All bottles were stored upside-down at room temperature until analysis. Propane concentrations were determined using a gas chromatograph coupled with IRMS (Delta^plus^XP; Thermo Fisher Scientific) via a combustion furnace and a conflow interface (GC Combustion III; Thermo Fisher Scientific) as described above. Errors were calculated as described by Julien et al. (37).

### Cracking of Long-Chain *n*-Alkane (*n*-C_25_).

Thermogenic propane was prepared by cracking commercial *n*-C_25_ (TCI Co.). The sample used is that described in Gilbert et al. (38). Around 1 mg of *n*-C_25_ was added to a Pyrex tube containig Pd wires previously treated with H_2_. The tube was then evacuated and sealed under vacuum. The tube was heated to 500 °C in a muffle furnace for 0.5 to 5 h at constant temperature. Molecular and intramolecular δ^13^C values of propane thus formed was measured as described above. Propane concentrations ranged from 4 to 15% of the volatile hydrocarbons, the other hydrocarbons being methane (39 to 46%) and ethane (47 to 51%).

### Calculation of Isotope Fractionation Factors from Theoretical Isotope Effects.

The relationship between δ^13^C_central_ and δ^13^C_terminal_ for thermogenic processes was calculated using theoretical fractionation factors (13). Propane formation from thermal cracking of a longer-chain alkane (“primary cracking”) was considered to occur through the formation of ^˙^CH_2_-CH_2_-CH_3_ radical, corresponding to the reaction

$$R–CH_{2}–CH_{2}–CH_{3}\to ˙CH_{2}–CH_{2}–CH_{3}.$$

Position-specific isotope composition of propane thus generated can be calculated as follows:

$${\delta C}_{\text{term}}={\delta C}_{0}-\varepsilon_{\text{term}}\frac{\left(1-F\right)\ln\left(1-F\right)}{F},$$ [8]

where δ^13^C_term_ is the isotopic composition of one of the terminal C-atom position of propane, δ^13^C_0_ is the isotopic composition of the starting material from which propane is evolved, ε_term_ is the fractionation factor associated with terminal C-atom position of evolved propane, and *F* is the extent of the reaction, namely the amount of propane formed relative to the starting material (13). The same equation can be derived for the central C-atom position as well as the second terminal position of propane.

Propane degradation through thermogenic reaction (“secondary cracking”) was considered to occur through the formation of ^˙^CH_2_-CH_3_ and ^˙^CH_3_ from propane:

$$CH_{3}–CH_{2}–CH_{3}\to ˙CH_{2}–CH_{3}$$

$$CH_{3}–CH_{2}–CH_{3}\to ˙CH_{3}.$$

The δ^13^C value of the terminal C-atom positions of propane consumed through thermogenic cracking reaction can be calculated as follows:

$${\delta C}_{\text{term}}={\text{δC}}_{0}-{\text{ε}}_{\text{term}}\ln\left(1-F\right).$$ [9]

Carbon isotope fractionation factors ε were those calculated in ref. 13 at four different temperatures. For each temperature, the slope of the relationship δ^13^C_central_ and δ^13^C_terminal_ vs. δ^13^C_propane_ was calculated. The value used in Fig. 1 is the average of the slopes obtained for four temperatures.

## Supplementary Material

Supplementary File

## References

[1] Hodnebrog Ø, Dalsøren SB, Myhre G (2018). Lifetimes, direct and indirect radiative forcing, and global warming potentials of ethane (C2H6), propane (C3H8), and butane (C4H10). Atmos Sci Lett.

[2] Simpson IJ (2012). Long-term decline of global atmospheric ethane concentrations and implications for methane. Nature.

[3] Etiope G, Ciccioli P (2009). Earth’s degassing: A missing ethane and propane source. Science.

[4] Dalsøren SB (2018). Discrepancy between simulated and observed ethane and propane levels explained by underestimated fossil emissions. Nat Geosci.

[5] Tzompa-Sosa ZA (2017). Revisiting global fossil fuel and biofuel emissions of ethane. J Geophys Res Atmos.

[6] Helmig D (2016). Reversal of global atmospheric ethane and propane trends largely due to US oil and natural gas production. Nat Geosci.

[7] Widdel F, Grundmann O, Timmis KN (2010). Biochemistry of the anaerobic degradation of non-methane alkanes. Handbook of Hydrocarbon and Lipid Microbiology.

[8] Aeckersberg F, Bak F, Widdel F (1991). Anaerobic oxidation of saturated hydrocarbons to CO2 by a new type of sulfate-reducing bacterium. Arch Microbiol.

[9] Kniemeyer O (2007). Anaerobic oxidation of short-chain hydrocarbons by marine sulphate-reducing bacteria. Nature.

[10] Quistad SD, Valentine DL (2011). Anaerobic propane oxidation in marine hydrocarbon seep sediments. Geochim Cosmochim Acta.

[11] Etiope G (2009). Evidence of subsurface anaerobic biodegradation of hydrocarbons and potential secondary methanogenesis in terrestrial mud volcanoes. Mar Pet Geol.

[12] Meng Q (2017). Gas geochemical evidences for biodegradation of shale gases in the Upper Triassic Yanchang Formation, Ordos Basin, China. Int J Coal Geol.

[13] Tang Y, Perry JK, Jenden PD, Schoell M (2000). Mathematical modeling of stable carbon isotope ratios in natural gases. Geochim Cosmochim Acta.

[14] Gilbert A, Yamada K, Suda K, Ueno Y, Yoshida N (2016). Measurement of position-specific ^13^C isotopic composition of propane at the nanomole level. Geochim Cosmochim Acta.

[15] Hinrichs K-U (2006). Biological formation of ethane and propane in the deep marine subsurface. Proc Natl Acad Sci USA.

[16] Tissot BP, Welte DH (1978). Petroleum Formation and Occurence: A New Approach to Oil and Gas Exploration.

[17] Piasecki A (2018). Position-specific ^13^C distributions within propane from experiments and natural gas samples. Geochim Cosmochim Acta.

[18] Rooney MA, Claypool GE, Moses Chung H (1995). Modeling thermogenic gas generation using carbon isotope ratios of natural gas hydrocarbons. Chem Geol.

[19] Jaekel U, Vogt C, Fischer A, Richnow H-H, Musat F (2014). Carbon and hydrogen stable isotope fractionation associated with the anaerobic degradation of propane and butane by marine sulfate-reducing bacteria. Environ Microbiol.

[20] Widdel F, Rabus R (2001). Anaerobic biodegradation of saturated and aromatic hydrocarbons. Curr Opin Biotechnol.

[21] Sherwood Lollar B, Weise SM, Frape SK, Barker JF (1994). Isotopic constraints on the migration of hydrocarbon and helium gases of southwestern Ontario. Bull Can Pet Geol.

[22] Clark ID (2015). Paleozoic-aged microbial methane in an Ordovician shale and carbonate aquiclude of the Michigan Basin, southwestern Ontario. Org Geochem.

[23] Hobbs MY, Frape SK, Shouakar-Stash O, Kennell LR (2011).

[24] Legall FD, Barnes CR, MacQueen RW (1981). Thermal maturation, burial history and hotspot development, Paleozoic strata of southern Ontario–Quebec, from conodont and acritarch colour alteration studies. Bull Can Pet Geol.

[25] Dollar PS, Frape SK, McNutt RH (1991).

[26] Oren A (2011). Thermodynamic limits to microbial life at high salt concentrations. Environ Microbiol.

[27] Martini AM (2003). Microbial production and modification of gases in sedimentary basins: A geochemical case study from a Devonian shale gas play, Michigan Basin. AAPG Bull.

[28] Giunta T (2019). Methane sources and sinks in continental sedimentary systems: New insights from paired clumped isotopologues ^13^CH_3_D and ^12^CH_2_D_2_. Geochim Cosmochim Acta.

[29] Boreham CJ, Edwards DS (2008). Abundance and carbon isotopic composition of neo-pentane in Australian natural gases. Org Geochem.

[30] Liu C, McGovern GP, Liu P, Zhao H, Horita J (2018). Position-specific carbon and hydrogen isotopic compositions of propane from natural gases with quantitative NMR. Chem Geol.

[31] Adams MM, Hoarfrost AL, Bose A, Joye SB, Girguis PR (2013). Anaerobic oxidation of short-chain alkanes in hydrothermal sediments: Potential influences on sulfur cycling and microbial diversity. Front Microbiol.

[32] Holler T (2009). Substantial ^13^C/^12^C and D/H fractionation during anaerobic oxidation of methane by marine consortia enriched in vitro. Environ Microbiol Rep.

[33] Ward JA (2004). Microbial hydrocarbon gases in the Witwatersrand Basin, South Africa: Implications for the deep biosphere. Geochim Cosmochim Acta.

[34] Sherwood Lollar B, Ballentine CJ (2009). Insights into deep carbon derived from noble gases. Nat Geosci.

[35] Edwards D, Zumberge J (2005).

[36] Hut G (1987).

[37] Julien M (2018). Expanded uncertainty associated with determination of isotope enrichment factors: Comparison of two point calculation and Rayleigh-plot. Talanta.

[38] Gilbert A, Yamada K, Yoshida N (2013). Exploration of intramolecular ^13^C isotope distribution in long chain *n*-alkanes (C_11_–C_31_) using isotopic ^13^C NMR. Org Geochem.

