# Supplementary material for: Intramolecular isotopic evidence for bacterial oxidation of propane in subsurface natural gas reservoirs
Source: Proc Natl Acad Sci U S A. 2019 Mar 18;116(14):6653–8. doi: 10.1073/pnas.1817784116 (PMC6452727; doi:10.1073/pnas.1817784116)
Supplement: Supplementary File [file pnas.1817784116.sapp.pdf]

Supplementary information

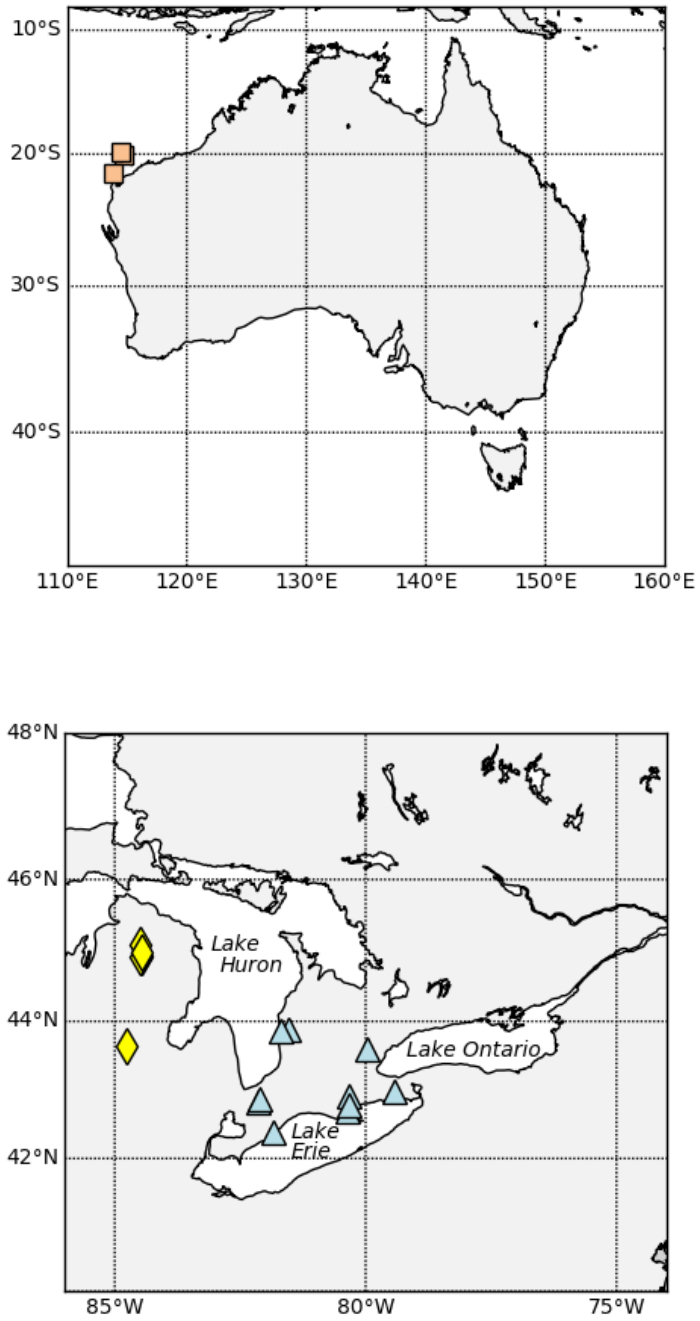

Figure S1. Geographical location of the samples analyzed in this work. Bottom: Southwest Ontario, Canada and Michigan Basin (USA). Top: Northern Carnarvon Basin (Australia).

www.pnas.org/cgi/doi/10.1073/pnas.1817784116

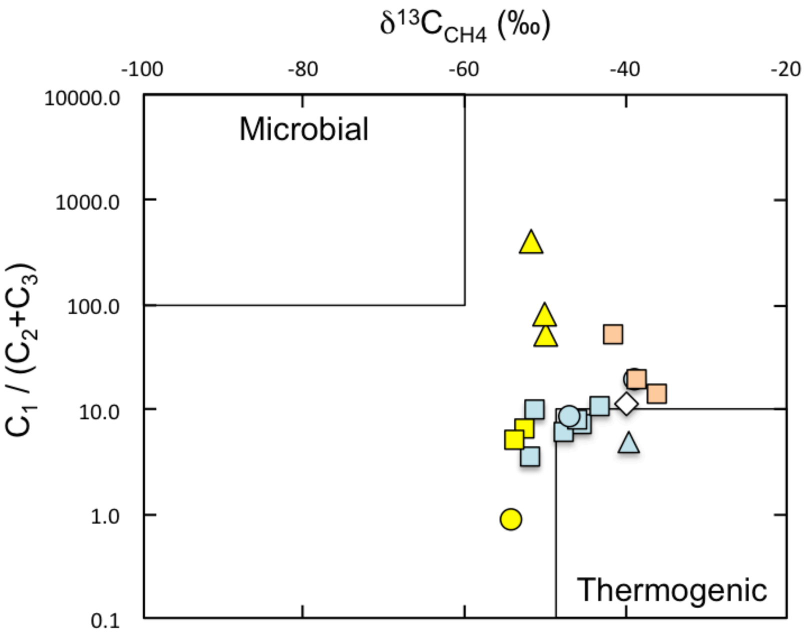

Figure S2. Carbon isotope composition of methane $\delta^{13}C_{CH4}$ plotted against its relative concentration C_1 / ($C_{2+}C_3$). Mixing of microbially-generated hydrocarbons with the thermogenic results in shifts in the data to more ^{13}C-depleted (more negative) δ^{13}C values and C_1 / ($C_{2+}C_3$) > 10. Symbols as in Figure 3. Error bars are smaller than the plotted symbols.

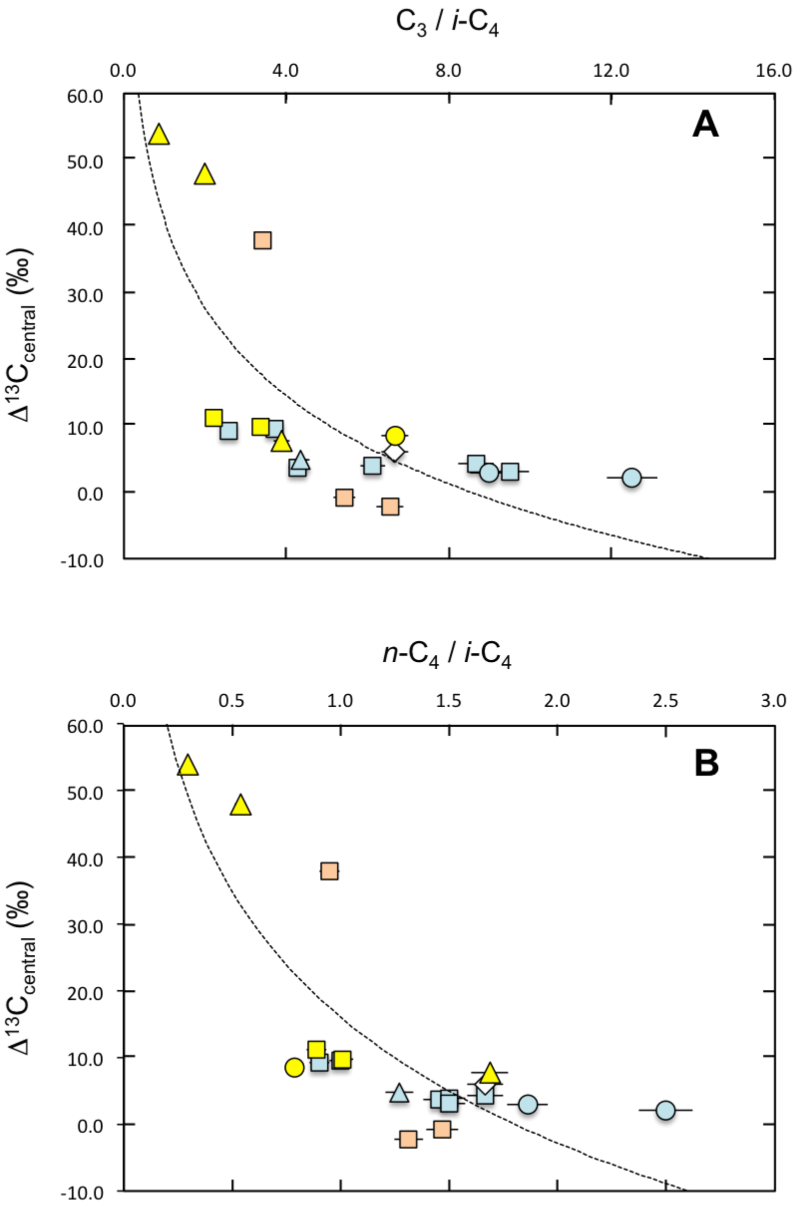

Figure S3. Relative ^{13}C-enrichment at the central C-atom position of propane ($\Delta^{13}C_{central}$) plotted against the relative chemical composition of propane (A) and *n*-butane (B) relative to *i*-butane (C_n/*i*-C_4) for the 19 samples from sedimentary basins. Lines represent logarithmic correlations with a significance > 95%. The relative concentration was calculated assuming *i*-butane (*i*-C_4) was not affected by biodegradation and was therefore constant. Symbols as in Figure 3.
